# Concomitant Use of Neuroprotective Drugs in Neuro Rehabilitation of Multiple Sclerosis

**DOI:** 10.4172/2329-9096.1000348

**Published:** 2016-06-23

**Authors:** Harika Dasari, Bharath Wootla, Arthur E Warrington, Moses Rodriguez

**Affiliations:** 1 Department of Neurology, Mayo Clinic, 200 First Street SW, Rochester, MN 55905, USA; 2 Mayo Clinic Center for Multiple Sclerosis and Autoimmune Neurology, Mayo Clinic, 200 First Street SW, Rochester, MN 55905, USA; 3 Department of Immunology, Mayo Clinic, 200 First Street SW, Rochester, MN 55905, USA

**Keywords:** Multiple sclerosis, Monoclonal antibodies, Neurorehabilitation, Physiotherapy, Neuroregenration

## Abstract

We provide an overview of rehabilitation in neurological diseases. A large amount of literature available on neurorehabilitation is based from the rehabilitative work on stroke and spinal cord injuries. After a brief description of rehabilitation, the potential application of neurorehabilitation in neurodegenerative diseases specifically multiple sclerosis (MS) is summarized. Since MS causes a wide variety of symptoms, the rehabilitation in MS patients may benefit from an interdisciplinary approach that encloses physiotherapy, cognitive rehabilitation, psychological therapy, occupational therapy, and other methods to improve fatigue. Neurorehabilitation helps patients to reach and maintain their optimal physical, psychological and intellectual, levels but it does not reverse long-term disabilities that arise from neurological disorders. This calls for the need of better neuroregenerative and neuroprotective treatment strategies in addition to neurorehabilitation. We discuss neuroprotective drugs aimed at preventing axonal, neuronal, myelin and oligodendrocyte damage and cell death that are approved and others that are currently in clinical trials, with an emphasis on human derived natural antibodies with remyleination potential. Our investigative group developed recombinant natural human IgM antibodies against oligodendrocytes and neurons with a potential for CNS repair and remyleination. One such recombinant antibody, rHIgM22 completed a phase 1 clinical trial with no toxicity and with an objective of promoting remyleination in multiple sclerosis. Inclusion of these drugs as a multifaceted approach may further enhance the efficacy of neurorehabilitation in neuroinflammatory and neurodegenerative disorders.

## Introduction

“Rehabilitation of people with disabilities is a process aimed at enabling them to reach and maintain their optimal physical, sensory, intellectual, psychological and social functional levels. Rehabilitation provides disabled people with the tools they need to attain independence and self-determination.” This definition describes the aims of rehabilitation as given by the World Health Organization.

Neurorehabilitation is a composite process that aims to aid recovery from a nervous system insult and thus to minimize or compensate functional alterations that occur as a result. Neurorehabilitation is a collaborative approach that works with the skills and attitudes of a disabled person, and helps to promote their skills in order to work at the highest level of independence possible thus helping them adapt to new situations and become empowered for a successful community reintegration. Hence it is a holistic intervention that is patient focused and strives on patients and their family's active involvement.

Our review highlights the use of a novel treatment modality using monoclonal naturally occurring antibodies (Nabs) [1] that have been proven to be efficacious in diseased models of MS with minimal or no toxic effects. Adjunctive use of these monoclonal antibodies with neurorehabilitation to address MS based spasticity and its symptoms may further enhance the therapeutic potential with possible improvement in the quality of life by maximizing the ability of MS patients to function. This unique approach holds a potential for application in other neurodegenerative diseases like Parkinson's disease, Amyotrophic Lateral Sclerosis and Alzheimer's disease.

## Neurorehabilitation in MS

Multiple sclerosis (MS) is one of the most common chronic demyelinating inflammatory diseases that is often relapsing or progressing with in the white matter of central nervous system (CNS). It preferably affects young adults and encompasses a wide range of symptoms such as visual disturbances, weakness of lower limbs, spasticity, coordination difficulties, sensory impairment, tremors and ataxia. However, covert symptoms like bladder disturbances, fatigue, depression and cognitive impairment occur frequently in the early phase of the disease [2]. These symptoms eventually cause neurological disability affecting the activities involved in day to day life.

Over the past few years there has been advancement in pharmacological treatment modalities for multiple sclerosis, particularly the disease modifying therapies and monoclonal antibodies. However, a considerable number of patients with MS develop new lesions and disabilities over time in the disease course, calling for the need of a multidisciplinary treatment strategy inclusive of rehabilitative approach to address the disabilities that result from the disease.

The central goal in MS patient's reduction of the limitations of activity and thus achieving a high level of independence with increased quality of life [3]. The present therapies available for MS, focus on the decrease in frequency of acute episodes without much emphasis on the improval of long term disability caused by the progressive neurodegenerative phase of MS. The long term disability arising from the progressive neurodegenerative phase of MS can be approached with a multifaceted modality with inclusion of physiotherapy, psychological therapy, cognitive rehabilitation, occupational therapy, speech therapy, improvement of fatigue and coping programs [4].

## Multidisciplinary Rehabilitation in MS

Over the past decades a growing number of research studies addressing the effectiveness of neurorehabilitation have been performed mainly in stroke patients but also quite a few in MS. About 10 clinical trials focused on the multidisciplinary approach to neurorehabilitation in MS were identified in a recent Cochrane review [5], which emphasizes that inpatient rehabilitation is more likely prone to have short term effects on the activities and participation of MS patients but not on impairment. There is also significant evidence that supports the role of both inpatient and outpatient rehabilitative programs in improving the disability, bladder dysfunction, and participation, unfortunately these improvements subside with time and last for a maximum of at least 12 months [6]. Therefore, it is needed to repeat these multidisciplinary rehabilitative approaches frequently.

## Physiotherapy and Exercise

The role of physiotherapy in MS rehabilitation is to improve motor function, gait stability and walking capabilities, thus strengthening of physical fitness and endurance among patients. It includes methods based on proprioceptive neuromuscular stimulation as well as other approaches like equipment-supported training, constraint-induced movement therapy (CIMT), treadmill exercises and robot-assisted gait training [2] which could be subjective and vary from one another. An applicable method is decided which is tailored in accordance with the disabilities and capabilities of the patient and available resources of the rehabilitation team. Physiotherapy is also intended to improve bladder disturbances and breathing dysfunction by training pelvic floor and respiratory muscles respectively [7,8].

In numerous studies even though the data is limited (small samples, varying interventions) there is considerate evidence on improving muscle weakness [9,10], balance [11], mobility [12], depression and fatigue [13]. These interventions are well tolerated with no associated side effects, positively influencing limitations due to disease and deconditioning effects of inactive lifestyle [14].

## Cognitive Rehabilitation

Cognitive dysfunction usually occurs early in the disease affecting social life and daily living [15]. Most commonly cognitive dysfunction is noted in the areas of information processing, attention span, memory and executive functions [16]. It is critical to recognize these changes early by appropriate neurological testing to address rehabilitative measures specifically addressing cognitive dysfunctions as drug treatments are not effective [17]. Treatment is focused on psychotherapy and neuropsychological training with the provision of aids (RIMS [18] Special training of attention, memory, executive functions and learning performances have shown benefits.

## Fatigue

Fatigue is a common debilitating symptom of MS; patients present with feelings of lassitude, abnormal tiredness, lack of energy and motivation that increases towards the end of the day impacting daily activities and work ability (RIMS [18]). Pathogenesis is still unknown and could be due to lesions of cortical or sub cortical pathways involving motor cortex and basal ganglia, autonomic dysfunction, hypothalamic pituitary adrenal axis dysregulation and endocrine disturbances [19]. Drug treatment is inefficient in treating fatigue related symptoms and thus needs to be managed by non-pharmacological measures like counselling, planning the day by taking regular breaks, energy management and specific neuropsychological training.

## Neurorehabilitation Clinical Trials in Multiple Sclerosis

A large number of studies are dedicated to dynamic rejuvenation of the motor system following acute events like stroke. Similar insults of motor system can also be noticed in chronic diseases such as MS, with a potential role of rehabilitative approach in improving the quality of life in MS patients. Brain activation is found to be exaggerated in MS patients with normal motor function compared to healthy controls, assessed by means of a finger tapping study [20]. Brain activation changes with increased diffuse brain injury and increased hand disability was found to be present in active as well as passive finger movements thus mirroring brain reorganization.

Zeller et al, attempted to expound the mechanisms of neuroplasticity in MS, by conducting a study of rapid onset central motor plasticity in 22 patients with moderately severe MS and comparing it to healthy controls by the use of paired associative stimulation (PAS), a protocol that assesses synaptic potentiation in the cerebral cortex in combination with repetitive nerve stimulation using trans cranial magnetic stimulation (TMS). MS patients were seen to worse in both clinical and para clinical motor function tests but there was an enhancement of corticospinal excitability and training induced increments of motor performance identical to that of controls. On the basis of the above findings it was concluded that the early steps of neuronal plasticity are not likely to limit compensatory changes in MS and therefore there is a need for rehabilitation efforts on mechanisms supporting the late stages of motor learning [21].

Another study conducted by Sastre-Garriga et al. investigated 15 MS patients and 5 healthy controls by means of functional magnetic resonance imaging (fMRI). This cognitive rehabilitation approach included 15 computer-supported sessions and 5 non-computer-supported cognitive stimulation sessions. After 5 weeks of cognitive training, patients demonstrated a significant clinical improvement of neuropsychological performance, and this corresponded with increased brain fMRI activity in cerebellar areas of the brain [7].

A large amount of the evidence of rehabilitative approach for MS was derive from the studies conducted in patients of stroke or spinal cord injuries (SCI). Electromyogram triggered neuromuscular stimulations Impairment oriented training, and robotic interactive therapies are some of the promising new rehabilitative techniques for neurological disorders [7].

## Drugs with Neuroprotective Potential

Multiple sclerosis is a chronic inflammatory neurodegenerative disorder. There is no treatment available to stop disease progression or reverse existing impairment. Almost every mechanism of damage in neurological diseases has been proposed as a therapeutic target for neuroprotection. Processes specific to the nervous system such as trophic factor signaling, axonal guidance, myelin formation and those specific to neurons such as apoptosis, energy supply, ionic balance are simulated with experimental therapies [22,23]. Regenerative therapies including stem cells may provide some neuroprotective effects by the release of trophic factors, suppressing local inflammation or by providing microenvironment for the survival of neurons, axons and oligodendrocytes [24]. Secondary neuroprotection can further be achieved by reduction of insult by restoring blood supply in ischemia, decreasing excitotoxicity by reduction of epileptogenic activity in seizures or decreasing CNS inflammation by using immunomodulatory drugs like galtiramer acetate, dimethyl fumarate or fingolimod (Table 1).

## Natural Antibodies

The existence of natural antibodies was met with initial skepticism, however work done by Avrameas [25,26] and Notkins [27,28] provided convincing evidence that NAbs are a part of human innate Ig repertoire [29]. Natural antibodies are of IgM, IgG and IgA isotypes. They are systemic surveillance molecules that help in recognizing damaged and stressed cells, invading pathogens and cellular debris for elimination by immune system. These natural antibodies are produced by innate B cells in a T cell independent manner. In humans, Nabs belong to IgM subtype and are secreted products of B1cells (CD20+, CD27+, CD 43+ and CD70-). They prevent immunity by enforcing B-cell central tolerance induction [30].

Nabs are present in the serum of healthy individuals in the absence of antigenic stimulation by foreign antigens. In our laboratory, we identified monoclonal IgM Nabs that recognize self-antigens on neural cells promoting remyelination from human serum and from B-cell lines obtained from healthy adult humans, fetal cord blood and patients with high immunoglobulin concentrations (i.e., multiple myeloma, waldenström's macroglobulinemia and monoclonal gammopathy of unknown significance). One such Nab, recombinant human monoclonal IgM antibody (rHIgM22) that specifically binds to live oligodendrocytes and myelin was recently evaluated in a 16-site phase 1 clinical trial [31] in 72 MS patients, with demonstration of the safety of antibody at all dose increments.

The discovery of CNS remyelination potential of Nabs by our group [32,33] was a major breakthrough. Prior to our discovery antibodies against myelin components in healthy animals were expected to be detrimental in those with active demyelinating disease. However we found that, all treated animals with Nabs showed a considerable endogenous remyelination in the demyelinating lesions. We then screened human sera with high immunoglobulin concentrations for natural antibodies. Serum samples were first screened for binding with live cerebellar tissue slices and then for live oligodendrocytes. Using this method we identified 2 natural IgM antibodies (sHIgM22 and sHIgM46) with remarkable remyelination *in vivo*. The sHIgM22 was cloned to generate recombinant rHIgM22 [34,35].

## Mechanism of Action for Nabs with Remyleination Potential

### Polyreactive nature of antibodies

Antibodies capable of promoting remyleination are polyreactive due to their flexible antigen-binding site. Most of the antibodies recognize multiple glycosylated sphingolipids with a ceramide or sphingosine backbone and essential membrane domain components, the hydrophilic carbohydrate moiety of these lipids is present at the cell surface and hence detectable by Nabs. These antibodies have very low dissociation constants. The tight binding of remyelinating antibodies to their oligodendrocyte targets may be the basis for their therapeutic potential [35].

### Access across blood brain barrier

The presence of blood brain barrier (BBB) raises the concern for the treatment of CNS disorders with antibodies and larger molecules. This is because the BBB controls the transport of hydrophilic substances like antibodies from the periphery into the CNS and hence it is a major obstacle for the brain and spinal cord delivery of peripherally injected antibodies. However, following injury of the CNS, the BBB is usually breached due to damage to the astrocytes resulting in leakage of components like platelets, monocytes, neutrophils, and macrophages from the blood into the lesion. The extent of BBB leakage in neuroinflammatory diseases is greater than that seen in neurodegenerative diseases. Dysfunction of the BBB is a hallmark of MS [36] seen in both chronic progressive MS [37] and acute plaques [38]. Crossover of antibodies can be increased by targeting receptors on BBB for transcytosis or active transport from blood into the CNS. This approach increases the penetration of antibody by 2-3% which is sufficient to convert a clinically ineffective antibody to an effective one [39].

Proof of therapeutic IgM crossing the BBB can be obtained from magnetic resonance imaging with the use of paramagnetic iron oxide particles [40]. *In vivo* localization of rHIgM22 in demyelinating lesions of Theiler virus infected mice was done using 3D, T2 weighted magnetic resonance imaging. In a recently concluded phase 1 clinical trial of rHIgM22, the recombinant human IgM was detected in 14/14 patients on day 2 and in 5/12 patients of day 29 following administration of a single dose [41]. This finding supports the hypothesis of permeability of the BBB to large molecules like IgM in MS.

### Calcium influx

All the Nabs, which enhance remyleination cause calcium influx into the astrocytes, mature oligodendrocytes and immature oligodendrocytes [42]. The IgM induced effects on calcium influx in astrocytes and oligodendrocytes differ from each other on the basis of signaling mechanisms and as well as the calcium pools within each cells (calcium is stored in the endoplasmic reticulum of astrocytes but present extracellularly in oligodendrocytes). The signaling mechanism in oligodendrocytes is dependent on α-amino-3-hydroxy-5-methyl-4-isoxazolepropionic acid glutamate receptor, whereas calcium influx in astrocytes is by phospholipase C mediated generation of inositol triphosphate. The signaling complex in OPCs includes platelet derived growth factor α receptor, integrin αvβ3, and the Src family kinases Lyn are all responsible for rHIgM22-mediated actions [43]. Of note is that only mixed glial cultures consisting of astrocytes, OPCs, and microglial cells demonstrated observable rHIgM22-mediated OPC proliferation whereas isolated OPCs alone did not respond [44]. This data demonstrates that astrocytic or microglial cofactors either provide the required microenvironment or play a role in the stimulation of the proliferative response. Hence the glial secreted PDGF derived from astrocytes plays an important role in IgM stimulated OPC proliferation and remyleination.

### Neuron binding nabs stimulating neurite extension

The therapeutic effects of Nabs that target myelin and oligodendrocytes in demyelinating disease models encouraged our group to study monoclonal IgM antibodies with a capacity of binding to neuronal cells as probable treatment options in neurological insults (sHIgM12 and sHIgM22). These antibodies are different from those that promote remyleination with no effect on the extent of remyleination *in vivo* or on calcium influx *in vitro*, but these neuron binding antibodies play a role in the stimulation of neurite extension [45] and hence a recombinant of sHIgm12 was generated (rHIgM12) [46]. Binding of this rHIgm12 to the surface of neuronal cells induced reorganization of the membrane leading to the stimulation of a neurite outgrowth [47] rHIgM12 also played a role in stimulation of hippocampal neurite out growth [48]. Peripheral administration of rHIgM12 as a single bolus helped improve motor function in chronic demyelinating disease model of mice and also improved brainstem N-acetyl aspartate concentrations-considered a biomarker for neuronal health of spinal cord axons in a model of progressive MS [49,50]. We identified that polysialic acid attached to the neuronal cell adhesion molecule as an antigen for rHIgM12 [51,52]. Polysialic acid is present in abundance in developing brains and its early expression is significant for critical development events including but not limited to neuronal precursor migration, oligodendrocyte progenitor proliferation and axonal sprouting.

### Anti-LINGO-1-Ab (Li81) BIIB0033

The anti-LINGO-1 antibody (BIIB0033) is a potential treatment for MS and optic neuritis patients currently in clinical trials. Leucine-rich repeat and Ig domain containing NOGO receptor interacting protein-1 (LINGO-1) is a functional component of Nogo signaling complex that interacts with the ligand-binding Nogo-66 receptor. Lingo-1 is almost exclusively expressed in CNS neurons and oligodendrocytes during both embryonic and post-natal stages [53-57]. Anti-LINGO-1 antibody was shown to promote spinal cord remyelination in the EAE model. It has a potential to stimulate regrowth of the myelin sheath, which is damaged in MS CNS lesions.

#### Renew clinical trial

In April 2015, results from the Phase II clinical trial RENEW (Biogen) were published [58]. This trial tested efficacy and safety of anti-LINGO-1 in the treatment of acute optic neuritis (AON). RENEW was a double-blinded, randomized, placebo controlled parallel group study performed in subjects with first episode of AON to study the ability of anti-LINGo-1 to facilitate repair of optic nerve lesions by promoting axonal remyelination. A total of 82 participants with first episode of AON were either given 6 doses of Anti-LINGO-1, once every 4 weeks for 20 weeks in total or a placebo. RENEW trial studied the effects on remyelination by measuring the latency of nerve conduction between retina and the visual cortex using full field visual evoked potential (FF-VEP). Results showed a 34% improvement in the recovery of optic nerve latency compared to that of placebo in the per-protocol population, but this was not found to be statistically significant (p=0.0504), perhaps due to the small size study population.

#### Synergy clinical trial

This clinical trial by Biogen tested the efficacy and safety of anti-LINGO-1 in patients with relapsing remitting multiple sclerosis (RRMS) or with Secondary progressive multiple sclerosis (SPMS) [59]. A total of 416 study subjects received β-IFN 1a once weekly with placebo or series of 3, 10, 30, or 100 mg of anti-LINGO-1 per KG body weight. Anti-Lingo-1 was given monthly for 72 weeks. The main focus of this clinical study was to investigate improvement in cognitive functions. The primary endpoint of SYNERGY trial is a conglomerate change in neuro-physical and/or cognitive function and includes imaging biomarkers that investigate the potential of anti-LINGO-1 to repair MS lesions in the brain.

### GNbAC1

GNbAC1 is a humanized mAb directed against multiple sclerosis – associated retrovirus (MSRV) gene that encodes an envelope protein (Env): MSRV-Env. The antibody is a recombinant DNA –derived humanized mAb of the IgG4/κ isotype and contains human framework regions and the complementary determining regions of a parent murine antibody. GNbAC1 has a high affinity for the MSRV-Env extracellular domain to which it binds selectively activating a proinflammatory and autoimmune cascade as a result of its interaction with Toll-like receptor 4. Due to its inhibitory effects on the differentiation of oligodendrocyte progenitor cell (OPC) and its proinflammatory effects, MSRV-Env may play a role in MS pathogenesis. A 10-year blinded observational study showed that the presence of MSRV in the CSF of early MS patients was found to be associated with a considerable rate of relapse-unrelated unremitting disability and secondary progression of MS [60]. MSRV-Env domain is highly expressed in MS specifically in brain demyelinating lesions with no role in normal human physiology this could be the basis for it being a potential therapeutic target for MS. A number of preclinical and clinical trials that were performed to assess the efficacy of GNbAc1 in MSRV-Env induced EAE models of MS have showed an absence of safety risk and absence of antibody-dependent or complement induced cytotoxicity with a very low level of cross-reaction with human tissues. Results from clinical studies conducted in 33 healthy subjects and a long-term clinical study in 10 MS patients showed that GNbAC1 is well tolerated without induction of immunogenicity and with a pharmacodynamics response on MSRV biomarkers [61,62].

Other strategies currently in clinical phases include the use of trophic factor compounds (erythropoietin BN201), antioxidant compounds like the green tea extract epigallocatechin-3-gallate, ginkgo biloba extracts, biotin, dimethyl fumerate, olexosime, methylthioadenosine, semaphoring blockers (VX15/2503) and ion channel modulators like carbamacepin, phenytoin, lamotrigine, amiloride, riluzole (Table 1). All these drugs are yet to show efficacy in phase 3 trials and it is difficult to know how they can be integrated in MS therapy and neurorehabilitation.

## Conclusion

From a clinical standpoint we know that patients who undergo neuro rehabilitation improve but this effect might not be long lasting. New strategies need to be identified for neuroinflammatory and neurodegenerative diseases and therapeutic approaches need to be focused on actively stimulating brain lesion repair and rehabilitation. This approach helps bridge the gap between basic pathology and symptomatic improvement of neurological diseases. Treatment targeting the immune system does not prevent or reverse long-term disabilities. The future for remyleination therapies with Nabs looks promising. A very low amount of human antibody rHIgM22 is needed for stimulation of remyleination in mice and relatively open BBB during acute phases of the disease are encouraging and thus give hope for MS patients. Prospective treatment strategies for MS can be enhanced by including agents targeting the nervous system like Nabs that stimulate remyleination or other neuroprotective drugs. These approaches are best when included with inclusion of neurorehabilitation to address the long-term disabilities that occur with MS.

## Figures and Tables

**Table 1:** Neuroprotective drugs for multiple sclerosis.

Drug	Company	Route	Compound – Mechanism of action	Clinical trial
	Preclinical			
ER agonist	Karo bio AB	N/A	Small chemical-Estrogen receptor beta agonist neuroprotection	N/A
NDC-1308	ENDECE Neural	N/A	Estradiol analog- neuroprotection remyelination	N/A
Methylthioadenosine	Digna Biotech	Oral	Methyltransferases modulator-neuroprotection remyelination	N/A
NRP 2945	CuroNZ	N/A	Peptide- Neuroprotection	N/A
KPT-350	Karyopharm	Oral	Selective Inhibitor of Nuclear export (SINE)-Antioxidant Neuroprotection	N/A
	Clinical			
rHIgM22	Acorda	IV	nab-recombinant human IgM, Remyelination	Phase 1 NCT01803867
BIIB0033- Anti-LINGO1	Biogen Idec	IV	mAb-Lingo-1 Antagonist, Remyelination and neuroprotection	Phase 2 NCT01864148
RNS60	Revalesio	IV	Physically modified saline, Immunomodulation remyelination	Phase 2 NCT01714089
MN-166 Ibudilast	MediciNova	oral	Small molecule, immunomodulation neuroprotection	Phase 2 NCT01982942
GNbAC1	GeNeuro	IV	mAb-Immunomodulation remyelination	Phase 2a NCT01639300
VX15/2503	Vaccinex	IV	mAb-anti-semaphorin 4D neuroprotection remyelination	Phase 1 Nct01764737
EGCG-Epigallacatechin-gallate	Generic	oral	Green tea extract, antioxidant	Phase 2 NCT00525668
Lamotrigine	GlaxoSmithKline	oral	Small chemical, Sodium channel modulator neuroprotection	NCT01879527
MRF-008 Guanabenz	Myelin Repair Foundation	oral	Alpha-2 adrenergic receptor agonist, Remyelination	Phase 1 NCT02423083
Clemastine	Generic		Small chemical, remyelination	Phase 2 NCT02040298
BAF312	Novartis	oral	Siponimod S1P1 antagonist, Neuroprotection	Phase 2 RRMS NCT00879658
BN201	Bionure	IV	Neurotrophin agonist, Neuroprotection	Phase 1
Erythropoietin	Generic	IV	Human recombinant protein, Trophic factor Neuroprotection	Phase 3 NCT01962571
Diazoxide	Generic	oral	Potassium channel opener, mitochondrial channel modulator	Phase 2 NCT01428726
GSK1223249	GlaxoSmithKline	IV	mAb Anti-Nogo-A, Axonal regeneration	Phase 2 NCT01435993
MD1003-Biotin	Generic	oral	Vitamin, Carboxylases coenzyme, remyelination	Phase 3 NCT02220933
Riluzole	Generic	oral	Small chemical, sodium channel and NMDA modulator	Phase 2 NCT00501943
Minocycline	Generic	oral	Small chemical, anti-apoptotic and anti-oxidant	Phase 2 NCT01073813
FTY720 Fingolimod	Novartis	oral	S1P1 and S1P5 antagonist, neuroprotection	Approved NCT00355134
BG12-Dimethyl fumarate	Biogen Idec	oral	Hydroxycarboxylic acid receptor 2 agonist, antioxidant	Approved NCT00420212
